# Measuring social vulnerability to natural hazards at the district level in Botswana

**DOI:** 10.4102/jamba.v11i1.447

**Published:** 2019-05-06

**Authors:** Kakanyo F. Dintwa, Gobopamang Letamo, Kannan Navaneetham

**Affiliations:** 1 Environment Statistics Unit, Statistics Botswana, Gaborone, Botswana; 2 Department of Population Studies, University of Botswana, Gaborone, Botswana

**Keywords:** Botswana, District Social Vulnerability, place vulnerability, natural hazards, principal component analysis

## Abstract

Social vulnerability to natural hazards has become a topical issue in the face of climate change. For disaster risk reduction strategies to be effective, prior assessments of social vulnerability have to be undertaken. This study applies the household social vulnerability methodology to measure social vulnerability to natural hazards in Botswana. A total of 11 indicators were used to develop the District Social Vulnerability Index (DSVI). Literature informed the selection of indicators constituting the model. The principal component analysis (PCA) method was used to calculate indicators’ weights. The results of this study reveal that social vulnerability is mainly driven by size of household, disability, level of education, age, people receiving social security, employment status, households status and levels of poverty, in that order. The spatial distribution of DSVI scores shows that Ngamiland West, Kweneng West and Central Tutume are highly socially vulnerable. A correlation analysis was run between DSVI scores and the number of households affected by floods, showing a positive linear correlation. The government, non-governmental organisations and the private sector should appreciate that social vulnerability is differentiated, and intervention programmes should take cognisance of this.

## Introduction

The impacts of natural hazards are becoming more prominent in developing countries because of inadequate preparedness, adaptation and mitigation strategies. Social vulnerability to natural hazards has become a topical issue owing to the major role it plays in the formulation of disaster risk reduction strategies. Chen et al. (2013) state that, by adopting the human-centred vulnerability concept, the social vulnerability school of thought (Blaikie et al. 1994; Cutter, Boruff & Shirley 2003; Hewitt 1997) stresses that vulnerability is socially constructed and manifests with stratification and inequality among different groups of people and different places (p.169). As a result, vulnerability reduction requires understanding of the underlying social, economic and political context and then addressing the factors that increase risk and vulnerability (Chen et al. 2013). Füssel (2007) stressed the need for an integrated approach for climate change vulnerability assessment which would also consider non-climatic factors (Bizimana, Twarabamenye & Kienberger 2015). Vulnerability assessment in this regard is an important vehicle for disaster risk reduction and climate change adaptation

Statistics Botswana (2014:48) reports that in 2010 a total of 418 households and 1669 individuals were affected by floods in the Ngamiland, North West, Kgatleng and Ngwaketse districts. In 2012, a total of 329 households and 1756 individuals were affected by floods in Mahalapye sub-district alone. Literature elsewhere also shows that the interaction of flood hazards and the vulnerability of flood-hazard-prone areas have caused regional flood disasters (Hsieh 2014; Zhang et al. 2010). For countries to effectively reduce the effects of disasters (e.g. floods, among others), there is a need to understand and internalise factors contributing to social vulnerability to flood-hazard-prone areas (Liu & Liang 2014), among other hazard-prone areas.

Since the inception of the Social Vulnerability Index methodology by Susan Cutter in 1996 (Vulnerability of Place Model [VPM]), this approach has gained momentum, and it has been replicated and applied in many countries around the World (Bjarnadottir, Li & Stewart 2011; Chen et al. 2013; Dunno 2011; Garbutt, Ellul & Fujiyama 2015; Noriega & Ludwig 2012; Siagian et al. 2014; Zebardast 2013). It is evident from the aforementioned studies that the assessment of social vulnerability to natural hazards can be conducted either at household level or district level, depending on data availability at each level. Other important social vulnerability models that have not been operationalised in this study include the Livelihood Vulnerability Index (LVI) model developed by Hahn, Riederer and Foster (2009). The LVI model builds on the Sustainable Livelihood Approach (SLA) by Chambers and Conway (1992). The SLA was developed mainly for assessing the ability of households to withstand shocks such as epidemics or civil conflict, with limited focus on addressing issues of sensitivity and adaptive capacity to the impacts of climate change. Furthermore, the SLA presents a more holistic approach about what resources poor people utilise to earn a living (Krantz 2001; Scoones 1998). These resources are referred to as the human capital (knowledge, education and health), natural capital (land and biodiversity), social capital (participation in decision-making, collective representation, formal and informal groups, networks and connections), physical capital (infrastructure – roads, shelter, transport, etc.) and financial capital (wages, savings, credit, etc.). Hahn et al. (2009) note that the LVI model is a new approach for vulnerability assessment that integrates climate exposure and accounts for household adaptation practices needed to comprehensively evaluate livelihood risks resulting from climate change. The LVI model was constructed to estimate the differential impacts of climate change on communities in two districts of Mozambique. The LVI uses multiple indicators to assess exposure to natural disasters and climate variability, social and economic characteristics of households that affect their adaptive capacity and current health, food and water resource characteristics that determine their sensitivity to climate change impacts (Hahn et al. 2009:75). Another important model developed for assessing of social vulnerability is that of Vincent (2004) which is called Index of Social Vulnerability to Climate Change for Africa (SVA). This model was constructed to empirically assess relative levels of social vulnerability to climate change-induced variations in water availability and permit cross-country comparison in Africa. In this study, a theory-driven approach was employed, that is, existing theoretical insights into the nature and causes of vulnerability to select variables for inclusion were used. The following variables were used to compute the Index of Social Vulnerability: economic well-being and stability; demographic structure; institutional stability and the strength of public infrastructure; global interconnectivity; and natural resource dependence. The shortfall of the model is that it was constructed at the national level. It would have been best if the model was applied at a district level, even to the smallest level (e.g. household level) in order to better inform decision-makers on where to focus their attention with regard to mitigation initiatives.

The VPM was operationalised in this study in an effort to compare vulnerability among different geographic locations (e.g. ^1^census districts of Botswana). The VPM was operationalised by exploring the social vulnerability component, and zeroing in on indicators that contribute to social vulnerability to natural hazards. The social vulnerability component of the VPM is best placed to help in the assessment of social vulnerability to natural hazards in Botswana. The merits of operationalising the VPM are that it can be used to compare vulnerability among different geographic locations, as well as the ability to analyse how social and biophysical factors interact spatially. Lastly, the VPM has the feedback mechanism that can be used to inform policy and mitigation-related strategies and programmes. The VPM has also guided several studies on vulnerability to natural hazards (Cutter & Finch 2008; Cutter, Mitchell & Scott 2000; Cutter et al. 2009; Dunno 2011).

The objectives of this study were to: (1) apply the Liu and Li’s household social vulnerability methodology of 2016 to measure district level social vulnerability to natural hazards in Botswana; (2) establish the key indicators contributing to social vulnerability to natural hazards in Botswana; and (3) compare social vulnerability between districts. It is important to undertake studies like this to provide scientific and empirical evidence that will guide disaster risk reduction strategies across census districts that are affected by natural extreme events and disasters.

## Materials and methods

The study used secondary data that were extracted from the 2011 Population and Housing Census of Botswana. This is because the census data had all the variables that were needed for analysis in this study. According to Statistics Botswana (2013), informed consent was obtained from individuals who voluntarily agreed to take part during the population and housing census.

### Derivation of data and measurement of variables

**FIGURE 1:**
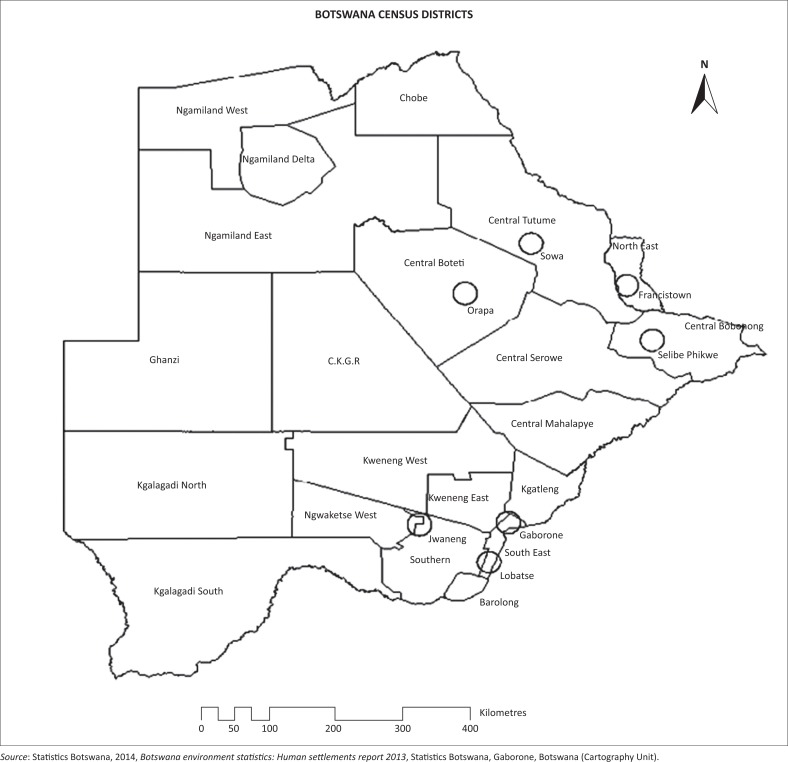
Botswana population and housing census districts, 2011. Census districts shown using circles are constituted by cities and mining towns.

Botswana comprises 28 census districts, which are constituted by administrative districts, cities and towns (Figure 1). A total of 28 census districts from the 2011 population and housing census data were used for the analysis (the unit of analysis is the census district). The selection of the study site was justified by the fact that most of the variables were available at census district level, and all the census districts have different levels of vulnerability which need to be investigated in order to inform mitigation and adjustment of related policies and programmes. All the people in the census districts are at risk of experiencing any disaster owing to their varying coping ability (demographic and socio-economic factors) and recovery from the impacts of natural hazard events, hence the need to investigate their level of social vulnerability.

A total of eight original variables of the Social Vulnerability Index Model developed by Cutter et al. (2003) were used in the development of the District Social Vulnerability Index (DSVI) model (see Table 1). A vigilant selection and rationalisation of the variables, with the intention of retaining only those most pertinent as indicators of social vulnerability, was done. Furthermore, the selection of the original variables was informed by the rule of thumb for PCA from Taylor’s analysis (1977), with the ratio of cases to variables set at 3.5:1. This ratio yielded eight variables to 28 study sites and/or cases. Three additional variables (see Table 2) were included in the list of original variables of the Social Vulnerability Index Model because they show communities’ preparedness prior to natural hazard events. They yielded a ratio of cases to variables = 2.6:1.

**TABLE 1:** Original social vulnerability variables used for analysis.

Variable name	Abbreviation code for PCA	Potential source
(1) Percent of population aged under 5 years	UNDER_FVE	2011 Population and Housing Census
(2) Percent of population aged over 65 years	OVER_SXTY	2011 Population and Housing Census
(3) Percent of unemployed civilian labour force	UNEMPLYD	2011 Population and Housing Census
(4) Average number of persons per household	NUM_HH	2011 Population and Housing Census
(5) Percent of population living in poverty (below $1.90 a day level)	PERC_POV	2009/2010 Botswana Core Welfare Indicator Survey
(6) Percent of population aged 25 years or older with no secondary education	NO_SEC	2011 Population and Housing Census
(7) Percent of households receiving social security	SOCSEC	2011 Population and Housing Census
(8) Percent of female-headed households (single parent)	FEMHEAD	2011 Population and Housing Census

PCA, principal component analysis.

**TABLE 2:** Additional social vulnerability variables used for analysis.

Variable name	Abbreviation code for PCA	Potential source
(1) Percent of households using any source of biomass for cooking (wood/charcoal and other methods)	BIOMAS_COOK	2011 Population and Housing Census
(2) Percent of people living with disability (partially/fully blind, hearing impaired, physically disabled and mentally retarded)	DISABLED	2011 Population and Housing Census
(3) Percent of people speaking a language at home other than Setswana	NO_SETS	2011 Population and Housing Census

PCA, principal component analysis.

Some of the additional indicators capture ethnicity and the cultural structure of Batswana. Botswana has a variety of ethnic groups living together within the same area. They gather together and socialise during certain events, like wedding ceremonies, funerals, *Kgotla* meetings and at workplaces. Each ethnic group holds on to its own culture (institutional practices, beliefs, values, norms and traditional religion). The variables added to best reveal the ethnicity and cultural composition of the Botswana census districts were: percent of households using any source of biomass for cooking (wood and/or charcoal and other methods); percent living with disabilities (partially or fully blind, hearing impaired, physically disabled and mentally retarded); and percent speaking a language at home other than Setswana or any Tswana-related dialects. The variable on the percent speaking a language at home other than Setswana was also used because it is an important component of ethnicity. The majority of the ethnic groups speak the Setswana Language as it is one of the two official languages in Botswana, other than the English Language (see Table 2). The use of electricity for either cooking or lighting is a preparedness variable in the sense that when electricity is down owing to floods or as an impact from any other natural hazard event, electricity on the main grid can be switched on after a short time as opposed to using biomass for cooking. Studies performed elsewhere support this, for example, Boruff and Cutter (2007) and Dunno (2011). Dunno (2011) adds that:

[…] although the Social Vulnerability Index is a valuable tool for measuring social vulnerability within a community, it should be necessary to adjust the original input variables so that they more accurately reflected the social fabric of the study area. (p. 51)

### Statistical methods

The District Social Vulnerability Index Model was computed because it quantifies the social vulnerability of different communities in a district to natural hazards, using social vulnerability indicators. It is based on the statistical method used in the Household Social Vulnerability Index (HSVI) methodology by Liu and Li (2016). The HSVI model does so by statistically assessing both the socio-economic and demographic factors influencing people’s capacity to cope and recover from environmental hazards.

### Data processing

After the data were collected, they were then normalised in order to create a standard dimension in the data set for ease of interpretation of the assessment results, though Liu and Li did not use the range 0–1. The data were normalised into a standard dimension. The standardisation was carried out using the following formula, for both positive and negative correlation indicators:

$$x_{i}^{\prime }=(x_{i}-minx_{i})/(maxx_{i}-minx_{i})$$ [Eqn 1]

where *x*_*i*_ and $x_{i}^{\prime }$ are the original and standard values of the indicator *i*, respectively. While *max x*_*i*_ and *min x*_*i*_ mean the greatest and the smallest values of the selected indicators in the data set.

### Weighting based on principal component analysis

For the determination of individual index weight, the PCA method was used with the help of SPSS software (version 22). An assessment of the weights was performed at a later stage by experts in the climate change field (disasters expert, environmental statistician, climatologist and climate change expert). Their assessment did not vary much from the PCA-based weighting; hence, the PCA-based weights were used as is (refer to sub-section ‘Calculation of weights’ for calculation of weights).

Prior to calculating the individual index weights, the following processes were undertaken.

#### Use of VARIMAX rotation and Kaiser criterion

The VARIMAX rotation was used because it has the potential to minimise the number of components. It also maximises the sum of variances of the components. The Kaiser criterion was also used as a factor selection option, keeping components with eigenvalues ≥ 1.0, as principal components.

#### Calculation of initial and rotated eigenvalues

The calculation of the initial and rotated eigenvalues was done, with particular reference to the initial eigenvalue, variance (%) and cumulative (%). In PCA, the value of cumulative (%) should be greater or equal to 80%. According to Liu and Li (2016), the cumulative (%) value ≥ 80% of the information of the extracted principal components could cover most of the information of the initial indictors (p. 1132). The calculation of the rotated component matrix values followed. The aforesaid values were used in the calculation of the PCA-based weights as shown in the following sub-section.

#### Calculation of weights

The calculation of PCA-based weights is an objective weighting method. The PCA-based weights of the individual index for the DSVI were calculated based on methodology as used in a study by Liu and Li (2016):

$$w_{i}=\frac{\sum_{j=1}^{k}\left(\frac{a_{ij}}{\sqrt{\lambda_{j}}}\times v_{j}\right)}{\sum_{i=1}^{n}\left[\sum_{j=1}^{k}\left(\frac{a_{ij}}{\sqrt{\lambda_{j}}}\times v_{j}\right)\right]}i=1,2,3\ldots n^{th},j=1,2\ldots n^{th}$$ [Eqn 2]

where *a*_*ij*_ is the value of the *i* th indicator at the *j* th rotated principle component and *λ*_*j*_ and *v*_*j*_ are the values of total initial eigenvalue and variance (%) at the *j* th rotated principle component, respectively (Liu & Li 2016:1132). The weights of the indicators included in this methodology can then be obtained from the above equation as follows:

$$w=[w_{1},w_{2},w_{3},\ldots w_{n^{th}}]$$ [Eqn 3]

The DSVI can be expressed by:

$$DSVI=\sum_{i=1}^{n}x_{i}\times w_{i},$$ [Eqn 4]

where *DSVI* is the District Social Vulnerability Index and *x*_*i*_ and *w*_*i*_ are standardised data and weight of the *i* th indicator, respectively.

### Mapping of the District Social Vulnerability Index scores

A comparative assessment of vulnerability using Geographic Information System (GIS) ARC INFO 10.2 mapping software was performed. Spatial data were obtained from the 2011 census. The DSVI scores were mapped using standard deviations (SD) as the classification algorithm to highlight the extremes (low and high) in social vulnerability in the study area. The study areas with low vulnerability (< −0.5 SD from the mean) and high vulnerability (> 0.5 SD from the mean) were identified. This comparative measure allows one to visually or numerically see how similar or how different places are relative to each other (Letsie 2015:66), and therefore make the DSVI a comparative measure of vulnerability. The colour ramps for the index scores were categorised as follows:

(1) Dark red (high): vulnerability index scores exceeding 1.5 SD; (2) light red (medium high): vulnerability index scores (0.5 SD – 1.5 SD); (3) Grey (medium): vulnerability index sources (< 0.5 – −0.5 SD); (4) light blue (medium low): vulnerability index sources (< −0.5 SD – −1.5 SD); and (5) dark blue (low): vulnerability index scores (< −1.5 SD).

## Results

After PCA performance, three components with eigenvalues > 1.0 were extracted. The eigenvalues were calculated, showing a total initial eigenvalue of 8.916, and a variance of 5.000% for Component 1; 2.724% for Component 2; and 1.092% for Component 3 (see Table 3). Table 3 also shows a total cumulative percentage of 81.057%, which is higher than 80.000%. According to Liu and Li (2016), the value of cumulative (%) should be equal to or higher than 80.000%, which demonstrates that the information of the extracted principle components could cover most of the information of the initial indictors (p. 1132). The rotated component matrix using VARIMAX with Kaiser normalisation as the rotation method is shown in Table 4. The highest squared loadings for social vulnerability indicators in each principal component are highlighted in bold.

**TABLE 3:** Initial eigenvalues and rotated eigenvalues of principal component analysis performed on indicators.

Component	Initial eigenvalues			Extraction sums of squared loadings			Rotation sums of squared loadings		
	Total	Percentage of variance	Cumulative percentage	Total	Percentage of variance	Cumulative percentage	Total	Percentage of variance	Cumulative percentage
1	5.100	46.366	46.366	5.100	46.366	46.366	3.408	30.982	30.982
2	2.724	24.764	71.130	2.724	24.764	71.130	2.993	27.207	58.189
3	1.092	9.926	81.057	1.092	9.926	81.057	2.515	22.868	81.057

Note: Extraction method – principal component analysis.

**TABLE 4:** Rotated component matrix.†

Variable	Component		
	1	2	3
UNDER_FIV	−0.026	0.164	**0.919**
OVER_SXTY	0.126	0.228	**0.929**
UNEMPLYD	−0.016	**0.787**	0.436
NUM_HH	0.344	**0.661**	0.502
PERC_POV	0.304	**0.719**	−0.046
NO_SEC	**0.896**	0.359	0.101
SOCSEC	**0.913**	0.113	0.183
FEMHEAD	0.082	**0.855**	0.187
ALT_COOK	**0.876**	0.131	−0.094
DISABLED	0.666	**0.671**	0.052
NO_SETS	**0.572**	−0.018	−0.523

Note: Data set in bold shows variables with both significant component loadings (≥ 0.5) and those which loaded highly on multiple components.

Extraction method – principal component analysis; Rotation method – VARIMAX with Kaiser normalisation. Refer to Table 5 for full names of indicators and definitions.

Data set in bold shows variables with both significant component loadings (≥ 0.5) and those which loaded highly on multiple components.

† , rotation converged in five iterations.

The results of the PCA-based weights of the all indicators are shown in Tables 5 and 6. It is evident from Table 5 that the indicators with the highest contribution to district social vulnerability are number of persons per household (0.125), disability (0.115), percent with no secondary education (0.113), percent over 65 years (0.107), people receiving social security (0.100), percentage of households under social safety nets (0.1000), percentage of households headed by females (0.093), percent under 5 years (0.088) and percent living in poverty (0.081), in that order.

**TABLE 5:** Selected indicators and their weights, definitions and measurement.

Indicator	Weight	Definitions	Measurement
UNDER_FIV	0.088	The percentage of population aged 5 years and below	Percent under five
OVER_SXTY	0.107	The percentage of population aged over 65 years	Percent over 65
UNEMPLYD	0.1	The percentage of population who are not employed	Percent unemployed
NUM_HH	0.125	The average number of people per household	Average number per household
PERC_POV	0.081	The percentage of population living under US$1.90 a day (below poverty datum line)	Percent living in poverty
NO_SEC	0.113	The percentage of population with no secondary education	Percent with no secondary
SOCSEC	0.1	The percentage of households under social safety nets	Percent receiving social security
FEMHEAD	0.093	The percentage of households headed by females	Percent of female-headed households
BIOMASS_COOK	0.076	The percentage of households using any source of biomass for cooking other than those using electricity	Percent using biomass for cooking
DISABLED	0.115	The percentage of people living with all types of disability	Percent with disability
NO_SETS	0.003	The percentage of households with population not speaking Setswana and Tswana-related dialects	Percent not speaking Setswana and related languages

**TABLE 6:** The standard data and assessment of District Social Vulnerability Index results by census district.

District	UNDER_FVE	OVER_SXTY	UNEMPLOYED	NUM_HH	PERC_POV	NO_SEC	SOCSEC	FEMHEAD	ALT_COOK	DISABLED	NO_SETS	Actual scores	Normalised DSVI scores	Ranks
Ngamiland West	0.004	0.015	−0.011	0.071	0.043	0.168	0.156	−0.004	0.157	0.131	0.089	0.820	1.000	High
Kweneng West	0.003	0.013	−0.011	0.054	0.044	0.195	0.138	−0.003	0.155	0.092	0.067	0.748	0.900	High
Ngwaketse West	0.001	0.003	−0.013	0.052	0.011	0.166	0.181	−0.003	0.164	0.075	0.092	0.728	0.872	High
Ghanzi	0.003	0.008	−0.010	0.047	0.032	0.139	0.112	−0.003	0.108	0.110	0.111	0.657	0.772	Medium high
Central Tutume	0.010	0.039	−0.014	0.052	0.019	0.138	0.129	−0.003	0.130	0.085	0.068	0.652	0.766	Medium high
Kgalagadi North	0.001	0.005	−0.010	0.039	0.016	0.128	0.144	−0.003	0.111	0.095	0.116	0.643	0.753	Medium high
Kgalagadi South	0.002	0.005	−0.013	0.050	0.022	0.132	0.165	−0.003	0.137	0.082	0.032	0.611	0.709	Medium high
Central Boteti	0.004	0.012	−0.017	0.056	0.019	0.122	0.142	−0.003	0.139	0.093	0.035	0.601	0.694	Medium High
Central Mahalapye	0.008	0.032	−0.015	0.057	0.016	0.140	0.138	−0.003	0.120	0.088	0.002	0.582	0.667	Medium high
Central Kalahari Game Reserve	0.003	0.009	0.000	0.031	0.000	0.112	0.209	0.000	0.136	0.007	0.071	0.578	0.662	Medium high
Barolong	0.004	0.015	−0.015	0.054	0.013	0.136	0.116	−0.003	0.138	0.094	0.000	0.550	0.623	Medium high
Ngwaketse	0.008	0.037	−0.016	0.063	0.019	0.132	0.116	−0.003	0.107	0.083	0.000	0.547	0.618	Medium high
Okavango Delta	0.000	0.001	−0.001	0.000	0.012	0.133	0.061	−0.003	0.192	0.078	0.070	0.543	0.613	Medium high
Central Serowe Palapye	0.011	0.045	−0.012	0.053	0.019	0.122	0.126	−0.003	0.096	0.080	0.005	0.542	0.612	Medium high
North East	0.004	0.016	−0.016	0.049	0.016	0.108	0.085	−0.004	0.099	0.065	0.077	0.499	0.552	Medium
Central Bobonong	0.005	0.019	−0.016	0.049	0.022	0.122	0.115	−0.003	0.109	0.058	0.001	0.481	0.527	Medium
Ngamiland East	0.006	0.017	−0.016	0.062	0.026	0.094	0.083	−0.003	0.086	0.074	0.029	0.457	0.493	Medium
Kweneng East	0.015	0.047	−0.014	0.052	0.017	0.080	0.048	−0.003	0.101	0.046	0.011	0.401	0.415	Medium
Kgatleng	0.005	0.024	−0.011	0.050	0.017	0.091	0.052	−0.003	0.082	0.048	0.005	0.360	0.357	Medium low
Chobe	0.001	0.003	−0.007	0.032	0.000	0.067	0.037	−0.003	0.055	0.029	0.056	0.270	0.231	Medium low
South East	0.004	0.014	−0.012	0.043	0.018	0.039	0.028	−0.003	0.042	0.053	0.014	0.239	0.188	Medium low
Lobatse	0.001	0.003	−0.011	0.032	0.016	0.037	0.017	−0.003	0.092	0.042	0.010	0.237	0.185	Medium low
Francistown	0.005	0.008	−0.011	0.033	0.016	0.019	0.024	−0.003	0.060	0.008	0.048	0.206	0.143	Medium low
Orapa	0.000	0.000	−0.007	0.024	0.002	0.056	0.003	−0.002	0.080	0.029	0.019	0.206	0.143	Medium low
Sowa Town	0.000	0.000	−0.007	0.020	0.021	0.013	0.004	−0.002	0.085	0.000	0.038	0.172	0.095	Medium low
Selibe Phikwe	0.002	0.003	−0.009	0.032	0.012	0.037	0.016	−0.002	0.027	0.016	0.013	0.145	0.058	Low
Jwaneng	0.000	0.000	−0.006	0.025	0.006	0.010	0.000	−0.001	0.062	0.009	0.018	0.124	0.028	Low
Gaborone	0.009	0.014	−0.008	0.030	0.011	0.000	0.008	−0.002	0.000	0.010	0.033	0.104	0.000	Low

Note: For abbreviated indicators, refer to Table 5 for full descriptions.

DSVI, District Social Vulnerability Index.

**FIGURE 2:**
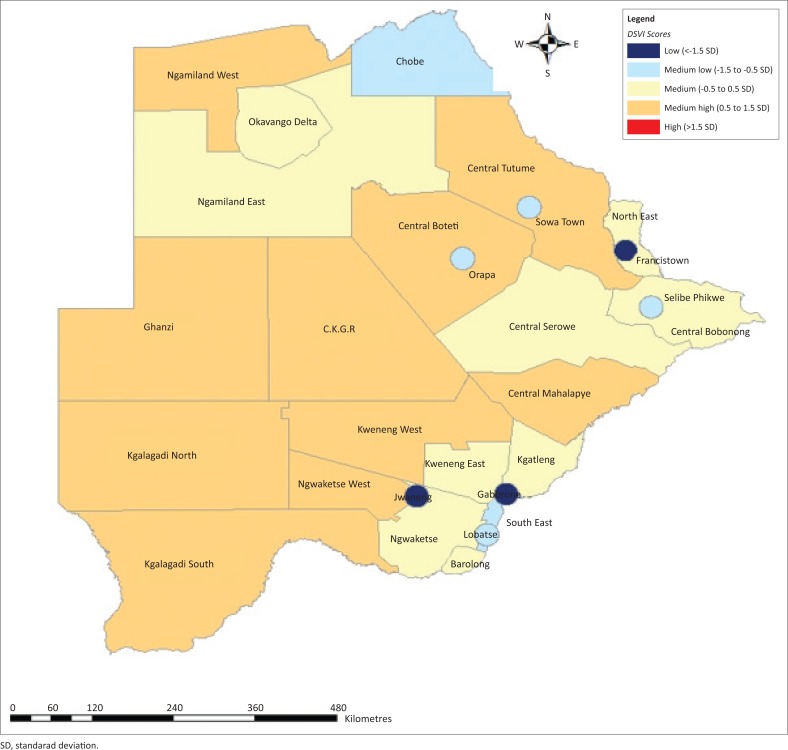
Spatial distribution of District Social Vulnerability Index scores by district in Botswana.

**FIGURE 3:**
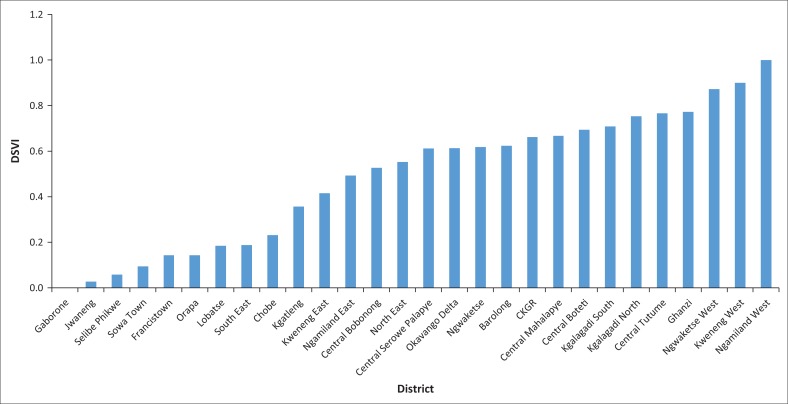
Ranked District Social Vulnerability Index scores from the lowest to the highest by district. DSVI, District Social Vulnerability Index.

For the spatial distribution of the DSVI scores of the districts, a mean of 0.488 and SD of 0.294 of the DSVI scores were used to divide the DSVI scores into high, medium high, medium, medium low and low/least vulnerability categories (see Table 6 and Figure 2). The district with the highest social vulnerability was Ngamiland West with a 1.000 DSVI score, while the lowest was Sowa Town and Orapa with 0.000 and 0.160 DSVI scores, respectively (normalised scores). The districts with the lowest social vulnerability are Gaborone (DSVI score, 0.000), Jwaneng (DSVI score, 0.028) and Selibe Phikwe (DSVI score, 0.058), in that order (Table 6 and Figure 3). Therefore, the results show that urban census districts are less vulnerable to natural hazards as compared to the urban–rural census districts. With regard to the proportion of each category on the total number of districts, it is evident from the results that the number of districts with high, medium high, medium, medium low and low vulnerability were 3, 11, 4, 7 and 3, respectively, constituting 10.7%, 39.3%, 14.3%, 25.0% and 10.7% of the total census districts, respectively.

A correlation analysis was run between DSVI scores and the number of households affected by floods to help to empirically provide evidence of the association between DSVI and the incidence of floods. The results reveal a positive linear correlation (*r* = 0.235) between DSVI scores and the number of households affected by floods. The *t*-test shows a 0.013 probability, implying that the correlation coefficient has a 98.7% probability of being true, and is significant at the 0.05 level (showing that the correlation is valid). However, there is still a need to run a correlation using long-term data to test for a correlation between the DSVI scores and the number of households affected by floods in order to get the robust results.

## Discussion

This study was conducted to establish the key indicators contributing to social vulnerability to natural hazards using census data at the district level, as well as to apply the Liu and Li’s household social vulnerability methodology to measure the district level social vulnerability to natural hazards in Botswana. Based on the weights of indicators of social vulnerability calculated using the PCA method and subsequent development of the DSVI model, it is reported that social vulnerability is mainly driven by the following indicators: size of household, disability, level of education, age, people receiving social security, employment status, household’s status and levels of poverty. In terms of gender being a predictor of social vulnerability, the result of this study is consistent with the study by Ajibade, McBean and Bezner-Kerr (2013) which reveals that gender coupled with income, occupation and health care access are predictors of social vulnerability. Fako and Molamu (1995) confirm that the majority of female-headed households are generally resource-poor; hence, they are unable to mobilise enough resources to cushion themselves against the impacts of drought. Another social vulnerability study by Muyambo, Jordaan and Bahta (2017) reveals that gender and external support contribute majorly to the social vulnerability of communal farmers to drought in O.R. Tambo District, and this is influenced by a huge imbalance in decision-making related to drought risk reduction. The cultural values of most African communities (e.g. Tswana, Sotho and Xhosa, among others) do not allow women to make decisions regarding the management of livestock; this is the sole responsibility of men.

This study also reveals that low education contributes to increased social vulnerability, which is in accordance with the findings of the studies conducted elsewhere (e.g. Rufat et al. 2015). Rufat et al. (2015) assert that:

… lower education coincides with poverty, over-crowding, unemployment, income inequality, and marginalization. Even if the poor and marginalized face fewer economic damage costs, the relative impact of damaging flood events are generally greater for low-income groups. It may take years for those who cannot afford the costs of repair, reconstruction, or relocation to recover from even a moderately damaging event (Masozera, Bailey & Kerchner 2007). (p. 474)

On the contrary, other studies (e.g. Muyambo et al. 2017) found out that the level of education contributes less to social vulnerability to natural hazards, and drought in particular. It was expected that the O.R. Tambo district which has high illiteracy levels would score high on social vulnerability. Instead, the study revealed that the respondents perceived indigenous knowledge as contributing to the slight resilience the farmers exhibited towards drought (Muyambo et al. 2017:5). Literature elsewhere (e.g. Dube & Sekhwela 2008; Motsholapheko, Vanderpost & Kgathi 2011) states that IKS in Botswana has helped improve the livelihoods of the communities, as well as adapt to the impacts of climate change.

The study also reveals that the high vulnerable population groups’ factor, which is mainly driven by high percent aged under 5 years, as well as high percent aged 65 years and above, contributes highly to social vulnerability to natural hazards in Botswana. The aforementioned population often lacks the ability to cope and recover from the impacts of natural hazard events as they often have low economic status and they are physically not able bodied enough to cope. This result is consistent with the results of studies conducted elsewhere; for example, Letsie and Grad (2015) found that ‘vulnerable population groups’ is one of the factors contributing to social vulnerability in the Mountain Kingdom of Lesotho. In Lesotho, the ‘vulnerable population groups’ factor is mainly driven by child-headed households, orphans and people aged 65 years or older.

It is for the aforementioned indicators that Ngamiland West,Kweneng West, Ngwaketse West, Ghanzi and Central Tutume have the highest DSVI scores, implying that they are highly socially vulnerable. According to the 2011 census data, there is high unemployment, high poverty levels and a high percentage of female-headed households, among others, in the aforementioned urban–rural census districts. These results are consistent with the findings of the study by Chen et al. (2013) which reveal that employment and poverty, education and family size are drivers of social vulnerability in China. Lack of employment exacerbates poverty levels, leaving the poor more prone and less resilient to the impacts of natural hazards. In an event where the unemployed and the poor are hit by floods, they cannot afford to rebuild houses that were destroyed by floods because of limited or a lack of financial resources. Furthermore, Cutter et al. (2003) also found that personal wealth, tenancy, vulnerability preparedness, ageing and social dependence, culture and social structure are the main factors influencing social vulnerability in the United States.

Disability was found to be one of the factors contributing to social vulnerability to natural hazards in Botswana. It is more pronounced in Ngamiland West, Ghanzi, Kweneng West and Kgalagadi North. These are rural–urban districts with almost the same socio-economic developments. There is high poverty and high unemployment, among other factors, in the aforesaid districts. The communities in these districts are mostly dependent on agriculture in order to sustain their livelihoods. Dunno (2011) notes that ‘it is a combination of economic status coupled with old age, and the potential for disability that causes a person to be more vulnerable’ (p. 133). Cutter et al. (2003) also add that populations with special needs, including people living with a disability, are disproportionately affected during disasters and, because of their invisibility in communities, are mostly ignored during rescue and recovery operations. Most of them lack the ability to communicate (language barrier for the deaf) and the ability to see (the blind), and these factors put them at high risk of being affected by the impacts of natural hazard events.

The results of this study also reveal that most of the urban census districts (cities and towns) are least socially vulnerable compared to the urban-rural districts. The majority of the population in the urban census districts are employed and can afford the basic necessities in life (food, clothing and shelter); therefore, recovering and coping with the impacts of natural hazard events would not be as difficult as that for the poor who reside in urban–rural census districts. This shows some imbalance in resource distributions between the urban and the rural populace. Chen et al. (2013) put forward some suggestions to reduce social vulnerability in the Yangtze River Delta region, such as reducing the unequal distribution of social resources and improving the employment rate (Chen et al. 2013; Liu & Li 2016:1128). The results of this study are therefore consistent with literature elsewhere, which states that medium to high socio-economic status enables communities to absorb and recover from losses more quickly owing to financial security (savings, insurance), social safety nets, decent jobs and entitlement programmes, among others (Cutter et al. 2000; Dunno 2011). Cutter et al. (2003) further add that poverty is the main driver of social vulnerability as fewer individual and community resources are available for recovery, thus making the poverty-stricken less resilient to the impacts of natural hazards. This justifies the results of this study that show that all the urban census districts (Gaborone, Francistown, Lobatse, Selibe Phikwe, Orapa, Sowa Town and Jwaneng) have medium low to low social vulnerability to natural hazards. The Liu and Li’s (2016) HSVI model is applicable in Botswana because it was able to appropriately compare vulnerability between census districts. Adjusting the HSVI model to include indicators that represent the local socio-economic structure of Botswana makes the HSVI model applicable to Botswana.

## Conclusion

This study expands Susan Cutter’s 1996 VPM by applying it in the middle to the high-income country of Botswana. An adjustment of the Model to best present the social fabric of the local environment shows the expansion of the model. It also applied Liu and Li’s (2016) HSVI methodology in Southern Africa, in Botswana in particular. Therefore, this study makes a theoretical and practical contribution to the body of literature on natural hazards and vulnerability. It does that by advancing both theory and knowledge regarding vulnerability within the context of a developing semi-arid region which is often faced with a lack of data on other important indicators contributing to social vulnerability to natural hazards.

Just like studies conducted elsewhere (e.g. Chen et al. 2013; Cutter et al. 2003; Dunno 2011; Letsie & Grab 2015), this study used census data, implying that the study can be updated as new data become available. This will allow for time series analyses of social vulnerability to natural hazards (Letsie & Grab 2015; Simpson & Katirai 2006).

Based on the PCA-weighted indicators of social vulnerability, it can be concluded that social vulnerability is mainly driven by the following indicators: size of household, people receiving social security, disability and level of education. There are high values of the aforementioned indicators in rural Botswana as compared to urban Botswana. This is attributable to the fact that economic development is more concentrated in the urban areas than in the rural areas. On the other hand, Ngamiland West, Kweneng West and Central Tutume have the highest DSVI scores, implying that they are highly socially vulnerable. Ngamiland West is ranked the highest in both the Social Vulnerability Index (SoVI) and District Social Vulnerability Index (DSVI) models. The districts with the lowest social vulnerability are Jwaneng, Orapa and Sowa Town, in that order. The correlation analysis was done between the DSVI scores and the number of households affected by floods to find out how correlated they are. The correlation coefficient of DSVI scores and the number of households affected by floods had a significance level of 0.05 (*r* = 0.235), with the two indicators having a positive correlation.

The analysis of the findings on the application of Liu and Li’s (2016) HSVI model to measure the district level social vulnerability to natural hazards in Botswana, informed the following key recommendations: The government should appreciate that social vulnerability is differentiated, thus intervention programmes should take cognisance of this. The increasing numbers of people living with a disability also calls for the government to increase its efforts in providing relevant education for people living with a disability, as well as employment opportunities, particularly in urban–rural districts. This effort will help people living with a disability to be more resilient and be able to cope with the impacts of natural hazard events. In terms of the government’s efforts to enhance natural hazards and disaster awareness in urban–rural census districts (particularly those prone to natural hazards), there is the need to: (1) intensify early warning systems by reaching the difficult-to-reach areas and improve the accuracy of the warning systems; (2) formulate and implement emergency management plans; and (3) increase the coverage of awareness-raising with regard to natural hazards and disasters.

### Limitations

The use of secondary data limits the research to indicators collected by the survey. Lack of data in other critical indicators of social vulnerability present yet another limitation, especially at the district level. Some of the important indicators had no data at the district level, for example, per capita number of community hospitals and per capita income. The use of PCA-based weights of indicators in developing the DSVI model has challenges. One of the challenges is the fact that the calculation of PCA-based weights needs a limited number of indicators, as many indicators present complex calculations and poor operativity (Cutter et al. 2003; Liu & Li 2016; Murphy & Scott 2014). As for this study, many indicators were used and this made the calculation of weights cumbersome. It was not possible to ground-truth the results of the DSVI model owing to a lack of funds.

## Footnotes

1 Census District: It means a geographic region defined for the purpose of undertaking a population and housing census. It sometimes coincides with the limits of cities, towns or other administrative areas/districts.

## References

[CIT0001] Ajibade I., McBean G. & Bezner-Kerr R, 2013, ‘Urban flooding in Lagos, Nigeria: Patterns of vulnerability and resilience among women’, *Global Environmental Change* 23(6), 1714–1725.

[CIT0002] Bizimana J., Twarabamenye E. & Kienberger S, 2015, ‘Assessing the social vulnerability malaria in Rwanda’, *Malaria Journal* 14, 2 10.1186/1475-2875-14-2 25566988 PMC4326441

[CIT0003] Bjarnadottir S., Li Y. & Stewart M.G, 2011, ‘Social vulnerability index for coastal communities at risk to hurricane hazard and a changing climate’, *Natural Hazards* 59, 1055–1075. 10.1007/s11069-011-9817-5

[CIT0004] Blaikie P., Cannon T., Davis I. & Wisner B, 1994, *At risk: Natural hazards, people’s vulnerability, and disasters*, Routledge, London.

[CIT0005] Boruff B.J. & Cutter S.L, 2007, ‘The environmental vulnerability of Caribbean Island nations’, *Geographical Review* 97(1), 932–942. 10.1111/j.1931-0846.2007.tb00278.x

[CIT0006] Chambers R. & Conway G, 1992, *Sustainable rural livelihoods: Practical concepts for the 21st century*, Institute of Development Studies, Brighton BN1 9RE, UK.

[CIT0007] Chen W., Cutter S.L., Emrich C.T. & Shi P, 2013, ‘Measuring social vulnerability to natural hazards in the Yangtze River Delta Region, China’, *International Journal of Disaster Risk Science* 4(4), 169–181. 10.1007/s13753-013-0018-6

[CIT0008] Cutter S.L, 1996, ‘Vulnerability to environmental hazards’, *Progress in Human Geography* 20(4), 529–539.

[CIT0009] Cutter S.L., Boruff B.J. & Shirley W.L, 2003, ‘Social vulnerability to environmental hazards’, *Social Science Quarterly* 84(1), 242–261. 10.1111/1540-6237.8402002

[CIT0010] Cutter S.L., Emrich C.T., Webb J.J. & Morath D, 2009, *Social vulnerability to climate variability hazards: A review of the literature*, Final Report to Oxfam America, Hazards and Vulnerability Research Institute, Columbia, South Carolina, pp. 1–44.

[CIT0011] Cutter S.L. & Finch C, 2008, ‘Temporal and spatial changes in social vulnerability to natural hazards’, *Proceedings of the National Academy of Sciences of the United States of America* 105(7), 2301–2306. 10.1073/pnas.0710375105 18268336 PMC2268131

[CIT0012] Cutter S.L., Mitchell J.T. & Scott M.S, 2000, ‘Revealing the vulnerability of people and places: A case study of Georgetown County, South Carolina’, *Annals of the Association of American Geographers* 90(4), 713–737.

[CIT0013] Dube O.P. & Sekhwela M.B.M, 2008, ‘Indigenous knowledge, institutions and practices for coping with variable climate in the Limpopo Basin in Botswana’, in Leary N. et al. (eds.), *Climate and adaptation*, pp. 71–89, Earthscan, London.

[CIT0014] Dunno C.H, 2011, ‘Measuring social vulnerability to natural hazards: An examination of the United States Virgin Islands’, PhD Thesis, University of North Carolina at Greensboro, Directed by Dr. Rick Bunch, pp. 1–207.

[CIT0015] Fako T.T. & Molamu L, 1995, ‘The seven-year drought, household food security and vulnerable groups in Botswana’, *Pula: Botswana Journal of African Studies* 9, 48–70.

[CIT0016] Füssel H.M, 2007, ‘Adaptation planning for climate change: Concepts, assessment approaches, and key lessons’, *Sustainable Sciences* 2, 265–275. 10.1007/s11625-007-0032-y

[CIT0017] Garbutt K., Ellul C. & Fujiyama T, 2015, ‘Mapping social vulnerability to flood hazard in Norfolk, England’, *Environmental Hazards* 14, 156–186. 10.1080/17477891.2015.1028018

[CIT0018] Hahn M.B., Riederer A.M. & Foster S.O, 2009, ‘The livelihood vulnerability index: A pragmatic approach to assessing risks from climate variability and change. A case study in Mozambique’, *Global Environmental Change* 19(1), 74–88. 10.1016/j.gloenvcha.2008.11.002

[CIT0019] Hewitt K, 1997, *Regions of risk: A geographical introduction to disasters*, Addison Wesley Longman Limited, London, England.

[CIT0020] Hsieh C.H, 2014, ‘Disaster risk assessment of ports based on the perspective of vulnerability’, *Natural Hazards* 74, 851–864. 10.1007/s11069-014-1214-4

[CIT0021] Krantz L, 2001, *The sustainable livelihood approach to poverty reduction: An introduction*, Swedish International Development Cooperation Agency, Division for Policy and Socio-Economic Analysis.

[CIT0022] Letsie M.M.A, 2015, ‘An assessment of place vulnerability to natural hazards in South-Western Lesotho (Quthing and Mohale’s Hoek districts)’, A thesis submitted to the Faculty of Science, University of the Witwatersrand, Johannesburg, in fulfilment of the requirements for the degree of Doctor of Philosophy, pp. 1–341.

[CIT0023] Letsie M.M. & Grab S.W, 2015, ‘Assessment of social vulnerability to natural hazards in the mountain Kingdom of Lesotho’, *Mountain Research and Development* 35(2), 115–126. 10.1659/MRD-JOURNAL-D-14-00087.1

[CIT0024] Liu B. & Li Y, 2016, ‘Social vulnerability of rural households to flood hazards in western mountainous regions of Henan province, China’, *Natural Hazards and Earth System Sciences* 16, 1123–1134. 10.5194/nhess-16-1123-2016

[CIT0025] Liu D.L. & Liang H.Q, 2014, ‘Social vulnerability assessment for regional natural disasters – A case study of He’nan province, Bull’, *Soil Water Conservation* 34, 128–134.

[CIT0026] Masozera M., Bailey M. & Kerchner C, 2007, ‘Distribution of impacts of natural disasters across income groups: A case study of New Orleans’, *Ecological Economics* 63(2), 299–306. 10.1016/j.ecolecon.2006.06.013

[CIT0027] Motsholapheko M.R., Vanderpost C. & Kgathi D.L, 2011, ‘Rural livelihoods and household adaptation to desiccation in the Okavango Delta, Botswana’, *Journal of Water and Climate Change* 3(4), 300–316. 10.1016/j.pce.2011.08.004

[CIT0028] Murphy E. & Scott M, 2014, ‘Household vulnerability in rural areas: Results of an index applied during a housing crash, economic crisis and under austerity conditions’, *Geoforum* 51, 75–86. 10.1016/j.geoforum.2013.10.001

[CIT0029] Muyambo F., Jordaan A.J. & Bahta Y.T, 2017, ‘Assessing social vulnerability to drought in South Africa: Policy implication for drought risk reduction’, *Jàmbá: Journal of Disaster Risk Studies* 9(1), a326 10.4102/jamba.v9i1.326 PMC6014179 29955328

[CIT0030] Noriega G.R. & Ludwig L.G, 2012, ‘Social vulnerability assessment for mitigation of local earthquake risk in Los Angeles County’, *Natural Hazards* 64, 1341–1355. 10.1007/s11069-012-0301-7

[CIT0031] Rufat S, Tate E, Burton C.G. & Sayeed Maroof A, 2015, ‘Social vulnerability to floods: Review of case studies and implications for measurement’, *International Journal of Disaster Risk Reduction* 14, 470–486. 10.1016/j.ijdrr.2015.09.013

[CIT0032] Scoones I, 1998, *Sustainable rural livelihoods: A framework for analysis*, Institute of Development Studies, Brighton.

[CIT0033] Siagian T.H., Purhadi P., Suhartono S. & Ritonga H, 2014, ‘Social vulnerability to natural hazards in Indonesia: Driving factors and policy implications’, *Natural Hazards* 70, 1603–1617. 10.1007/s11069-013-0888-3

[CIT0034] Simpson D.M. & Katirai M, 2006, *Indicator issues and proposed framework for a disaster preparedness index (DPi)*, Working Paper 06–03, Center for Hazards Research and Policy Development, University of Louisville, Louisville, KY.

[CIT0035] Statistics Botswana, 2013, *Environment statistics report 2012*, Department of Printing and Publishing Services, Government of Botswana.

[CIT0036] Statistics Botswana, 2014, *Botswana environment statistics: Human settlements report 2013*, Statistics Botswana, Gaborone, Botswana.

[CIT0037] Taylor C.C, 1977, ‘Principal component analysis and factor analysis’, in O’Muircheartaigh C.A. & Payne C. (eds.), in *Exploring data structures*, John Wiley & Sons, Inc., New York, pp. 89–124.

[CIT0038] Vincent K, 2004, *Creating an index of social vulnerability to climate change for Africa*, Tyndall Centre for Climate Change Research Working Paper 56, Tyndall Centre for CC Research, University of East Anglia, Norwich, UK.

[CIT0039] Zebardast E, 2013, ‘Constructing a social vulnerability index to earthquake hazards using a hybrid factor analysis and analytic network process (F’ANP) model’, *Natural Hazards* 65, 1331–1359. 10.1007/s11069-012-0412-1

[CIT0040] Zhang B., Yuan H.Y., Huang Q.Y., Wen R.Q. & Gu J.Q, 2010, ‘Research on fine spatial quantitative model about vulnerability of hazard-affected bodies’, *International Journal of Digital Earth* 3, 395–405. 10.1080/17538947.2010.496497

